# Welding Seam Recognition and Trajectory Planning Based on Deep Learning in Electron Beam Welding

**DOI:** 10.3390/s26020641

**Published:** 2026-01-18

**Authors:** Hao Yang, Congjin Zuo, Haiying Xu, Xiaofei Xu

**Affiliations:** 1 China Aviation Manufacturing Technology Research Institute, Beijing 100024, China; 18811158370@163.com (H.Y.); zuocongjin@126.com (C.Z.);; 2 School of Materials Science and Engineering, Nanjing University of Science and Technology, Nanjing 210094, China

**Keywords:** machine vision, deep learning, YOLOv11-seg, weld recognition, trajectory planning, electron beam welding

## Abstract

To address challenges in weld recognition during vacuum electron beam welding caused by dark environments and metal reflections, this study proposes an improved hybrid algorithm combining YOLOv11-seg with adaptive Canny edge detection. By incorporating the UFO-ViT attention mechanism and optimizing the network architecture with the EIoU loss function, along with adaptive threshold setting for the Canny operator using the Otsu method, the recognition performance under complex conditions is significantly enhanced. Experimental results demonstrate that the optimized model achieves an average precision (mAP) of 77.4%, representing a 9-percentage-point improvement over the baseline YOLOv11-seg. The system operates at 20 frames per second (FPS), meeting real-time requirements, with the generated welding trajectories showing an average length deviation of less than 3 mm from actual welds. This approach provides an effective pre-weld visual guidance solution, which is a critical step towards the automation of electron beam welding.

## 1. Introduction

As one of the indispensable technologies in aerospace processing, EBW technology with features of high energy density, low heat-affected zone, high weld depth-to-width, and pollution-free vacuum working space [1] has been regarded as an integral part. The manufacturing of critical parts like super alloy engine casings, titanium alloy blade, rocket engine thrust chamber, precise satellite brackets [2], etc., becomes inevitable with the EBW. The process advantages of EBW for welding reactive metals and high temperature alloys that are not readily achievable by the conventional welding process [3] have been shown.

However, most mass production EBW systems have basically been using the “offline programming high precision fixture” technique, which requires an extremely accurate absolute workpiece position [4] and is incapable of counteracting real-time dynamic errors from clamping deviation, welding thermal deformation, and material shrinkage [5]. The old ways to fulfill the small-lot, various models’ flexible manufacturing have their flaws: they teach path manually, align and detect welding paths, which not only prolongs the production preparation time, but also significantly decreases the production efficiency and the stability of welding quality. The magic pill to overcome these challenges and pave the way for smart evolution of EBW technique [6] is to achieve automatic weld recognition and navigation path planning.

Currently, mainly two kinds of the weld identification technology can be divided into conventional machine vision technology and deep learning. Wang et al. [7] believe that sensors for vision, structured light scanning, or morphological image processing can be commonly utilized to extract weld features to achieve detection of the welding quality. The study proved this traditional method could work well in a normal atmospheric environment. Zhao et al. [8]. utilized a light and improved model on the basis of DeepLabv3+ semantic segmentation network to achieve identification and weld trajectory planning. The image segmentation algorithm identified the image and then simulated the image trajectory with least squares method. YOLOv8-seg model was designed and integrated into the robot for accurate weld tracking. To achieve a higher real-time effect, TensorRT was used for acceleration ahead of deployment. Due to dark operation, high reflective metals, electrical and magnetic noise caused by electron guns, and metal vapor or dust etc., these approaches are also expected to encounter significant problems in EBW vacuum chambers [9]. The low image signals to noises and unstable feature extractions may become further critical technical barriers. The impressive capability of deep learning, especially object detection and segmentation networks, for welding applications have recently been demonstrated because of their effective feature learning and interference-resistant property. These networks can identify the weld position and geometry in spite of a highly complicated background [10]. Although the existing works are mainly focused on the arc welding or laser welding, there are only very few end-to end systems for industrial production developed for such welding applications with vacuum electron beam sources especially regarding high reflection materials, deep narrow weld geometry and optically impaired vision in vacuum.

In this paper, we design a mixed solution to these problems, taking the advantages of DL and traditional vision simultaneously. We fine-tuned YOLOv11-seg and integrated adaptive Canny edge detection to train an integration welding seam recognition and path planning framework. The translation between G-code and physical coordinates and the G-code generation provide a convenient way to realize the fusion of visual sensing and welding path generation. This paper aims at aiming automation from visual recognition of seams to trajectory planning and G-code generation to enable key technical support for intelligence augmentation of high end EBW equipment. To clearly articulate the methodological contributions of this work beyond system-level integration, the main innovations of this paper are summarized as follows:

1. Algorithmic Innovation for Challenging Environments: We propose a novel integration of the UFO-ViT attention mechanism into the YOLOv11-seg architecture.
2. Enhanced Detection for Specific Weld Geometries: To address the challenge of accurately detecting weld seams with extreme aspect ratios (long and narrow), we introduce optimizations in the detection head, including increasing the reg_max parameter and adjusting the positive sample IoU threshold.
3. A Hybrid “Coarse-to-Fine” Recognition Framework: We architect a robust two-stage framework that synergistically combines deep learning-based coarse segmentation (YOLOv11-seg) with traditional adaptive Canny edge detection.
4. An End-to-End System Integration from Vision to Motion: Beyond visual recognition, this research implements a complete closed-loop workflow from image acquisition and seam recognition to physical coordinate transformation and automated G-code generation.

## 2. Algorithm Implementation and Key Technologies

### 2.1. The Overall Framework of the Algorithm

**Figure 1:**
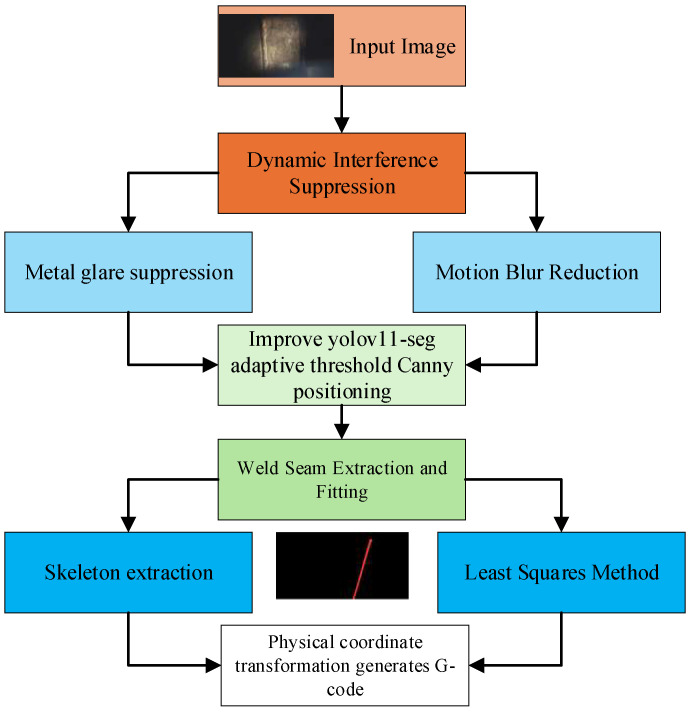
Flowchart of the algorithm’s overall framework.

In order to realize the robust identification of weld seam and high precision trajectory generation in a vacuum environment, the two-stage algorithm framework of “from coarse to fine” is designed in this paper, the flowchart is shown in Figure 1.

### 2.2. Improving the Coarse Positioning of Welds in YOLOv11-Seg

**Figure 2:**
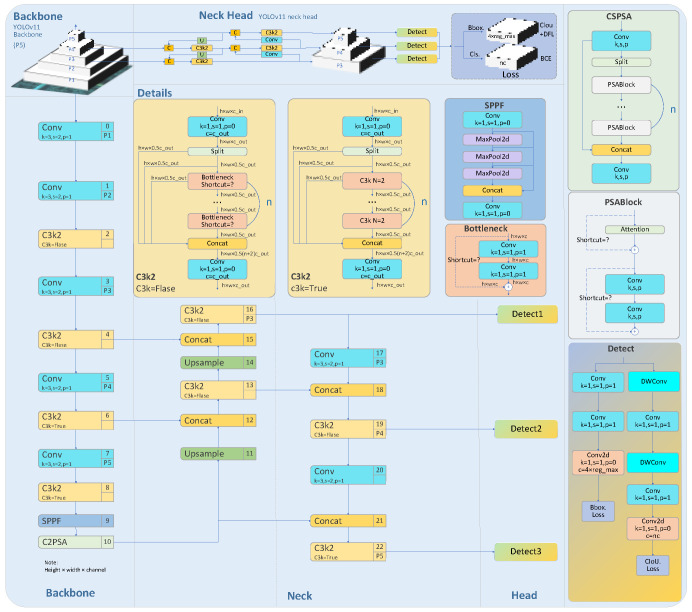
Network architecture diagram of YOLOv11-seg.

YOLOv11-seg was selected as the base model due to its excellent balance between speed and accuracy [11]. To accommodate diverse application requirements and resource constraints, YOLOv11 offers five model sizes—n, s, m, l, and x—where the model size and accuracy progressively increase [12]. The architecture of YOLOv11-seg is illustrated in Figure 2.

Two core optimizations are implemented for the task of weld recognition in a vacuum environment: (1) the introduction of the UFO—ViT attention mechanism; (2) the optimization of the loss function and the addition of a large-target detection head.

#### 2.2.1. Add Attention Mechanism

**Figure 3:**
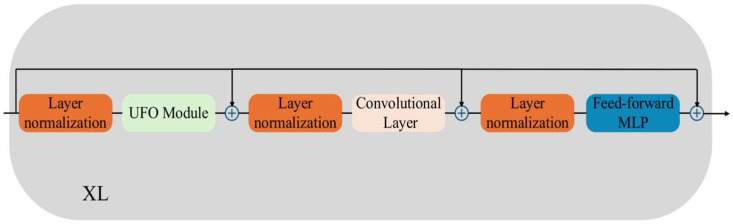
Schematic diagram of the UFO-ViT module structure.

This research enhances the original YOLOv11 model through the incorporation of an attention mechanism. Weld feature recognition demands a considerable amount of attention resources [13], which notably enhances the model’s ability to identify welds in vacuum environments. Nevertheless, the attention mechanism also elevates computational costs and operational burden. To address the challenge of weld groove extraction in complex scenarios, this research adopts the Unit Forced Operation Visual Transformer (UFO-ViT), a solution that neither substantially increases computational resource consumption nor requires additional computational resources. Figure 3 illustrates the structural diagram of the UFO-ViT module:

The UFO-ViT module leverages XNrom instead of softmax, thereby allowing the self-attention (SA) module to prioritize computational execution. XNrom is formally defined in Equations (1) and (2) as follows:

(1) $$A(x)=\bar{X}N_{dim}=filter(\bar{Q})(\bar{X}N_{dim=spase}(K^{T}V))$$

(2) $$XN(a)=\frac{ya}{\sqrt{\sum_{i=0}^{h}\|a{\|}^{2}}}$$

In the formula, γ stands for the learnable parameter, while h refers to the embedding dimension. It is a straightforward L2 norm applied to both the spatial and channel dimensions of q. When XNorm is used for both queries and outputs, it covers Formulas (3) to (5).

(3) $$A(x)=\left[\begin{array}{cccc} {\hat{q}}_{0}{\hat{k}}_{0} & {\hat{q}}_{0}{\hat{k}}_{1} & \ldots & {\hat{q}}_{0}{\hat{k}}_{n}\\ {\hat{q}}_{1}{\hat{k}}_{0} & {\hat{q}}_{1}{\hat{k}}_{1} & \ldots & {\hat{q}}_{1}{\hat{k}}_{n}\\ \vdots & \vdots & \ddots & \vdots \\ {\hat{q}}_{N-1}{\hat{k}}_{0} & {\hat{q}}_{N-1}{\hat{k}}_{1} & \ldots & {\hat{q}}_{N-1}{\hat{k}}_{n} \end{array}\right]$$

(4) $$q_{j}=XN\left[\left(Q_{0},Q_{1},\ldots Q_{d}\right)\right]$$

(5) $${\hat{k}}_{i}=XN\left[\left([K^{T}V{]}_{01},[K^{T}V{]}_{11},\ldots [K^{T}V{]}_{n1}\right)\right]$$

As shown in Equation (6), the projection weights—with x as the input—are computed via weighted sum, scaling, and dot product aggregation:

(6) $${\left[W_{pqr}A(x)\right]}_{k}=\sum_{n=1}^{h}w_{pq}^{\wedge }q_{i}^{\wedge }k_{j}^{\wedge }$$

In comparison to other conventional attention mechanisms, the UFO-ViT module delivers a batch processing capacity up to fourfold higher, surpassing most cutting-edge models based on Transformers and CNN architectures. Furthermore, it consumes fewer GPU resources.

#### 2.2.2. Optimization of Loss Function

The YOLOv11-seg model utilizes the Complete Intersection-Union (CIoU) loss function in bounding box regression tasks for assessing prediction quality, as outlined in Formulas (7)–(10):

(7) $$LOSS_{CLU}=1-IOU+\frac{p^{2}}{c^{2}}+\alpha v$$

(8) $$loU=\left|\begin{array}{c} A\cap B\\ A\cup B \end{array}\right|$$

(9) $$\nu =\frac{4}{\pi^{2}}{\left(\arctan\frac{\omega^{gr}}{h^{gr}}-\arctan\frac{\omega }{h}\right)}^{2}$$

(10) $$\alpha -\frac{\nu }{1-IoU+\nu }$$

This study uses the following notation: let A stand for the manually annotated position (real frame) and B signify the model-predicted position (predicted frame). The intersection area between these two frames is denoted as |A∩B|, while their union area is represented by |A∪B|—IoU; the intersection-over-union ratio between real and predicted frames is derived from these values. Additionally, ρ denotes the centroid distance between real and predicted frames, c signifies the diagonal length of the minimum enclosing rectangle, and v reflects the aspect ratio similarity between the frames (with α as its influence factor). Finally, wgt and hgt represent the width and height of the real frame, respectively, whereas W and H stand for those of the predicted frame.

The CIoU function boosts regression precision through centroid distance and aspect ratio constraints. Yet, its aspect ratio consistency loss shows slower convergence rates for targets with extreme aspect ratios (e.g., elongated shapes). To mitigate this issue, CIoU was adjusted and refined to create the EIoU function, which is formally defined as the loss function in Equation (11):

(11) $$L_{G\times U}=1-\bar{\sigma }U+\frac{\sigma^{2}(b,b^{\sigma })}{c^{2}}+\frac{\sigma^{2}(w,w^{\sigma })}{c_{w}^{2}}+\frac{\sigma^{2}(h,h^{\sigma })}{c_{h}^{2}}$$

To mitigate uncertainties during the optimization process, EIoU transforms the width–height penalty within CIoU’s implicit constraints into explicit computations. Meanwhile, the system dynamically modifies the penalty weight according to the relative positions between the ground-truth frames and the predicted frames, thus expediting the convergence of high-IoU samples.

#### 2.2.3. Optimization of Large Target Detection

**Figure 4:**
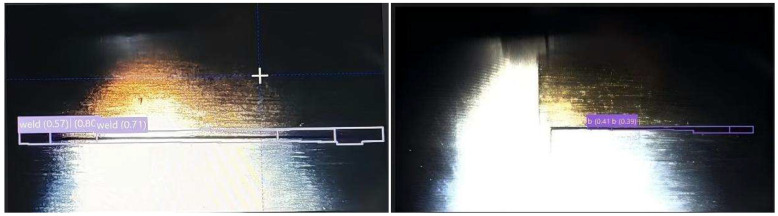
General model recognition performance.

For the horizontal long weld seam in the image occupying the pixel, due to the extreme length and width ratio characteristics, it is difficult for the anchor frame of the benchmark model to achieve accurate fitting, and incomplete positioning or repeated detection problems often appear [14], as shown in Figure 4.

To achieve this, the model incorporates an adaptive anchor bounding box clustering algorithm and raises the IoU threshold for positive sample allocation from 0.5 to 0.6, thereby enhancing its sensitivity to large object shape features. Additionally, the reg_max parameter of the regression branch in the detection head (Head) is significantly increased from 16 to 128. This parameter determines the granularity of distributed anchor prediction, with its modification Formula (12) as follows:

(12) $$B=\sum_{i=0}^{reg\_max}p_{i}\cdot i$$

**Figure 5:**
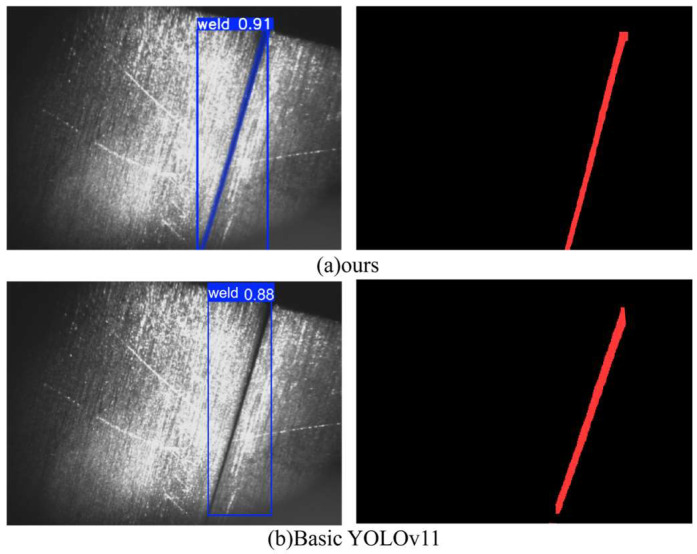
Comparison of the recognition performance of the improved model with that of the basic YOLOv11.

Here, B represents the predicted offset of the bounding box, and pi denotes the predicted distribution value of the model. Increasing reg_max enables the model to employ finer discrete distributions for bounding box localization, which significantly improves boundary regression accuracy for long weld targets. As shown in Figure 5, the optimized recognition achieves complete, continuous, and non-overlapping detection of long welds.

#### 2.2.4. Model Training and Validation Process

**Figure 6:**
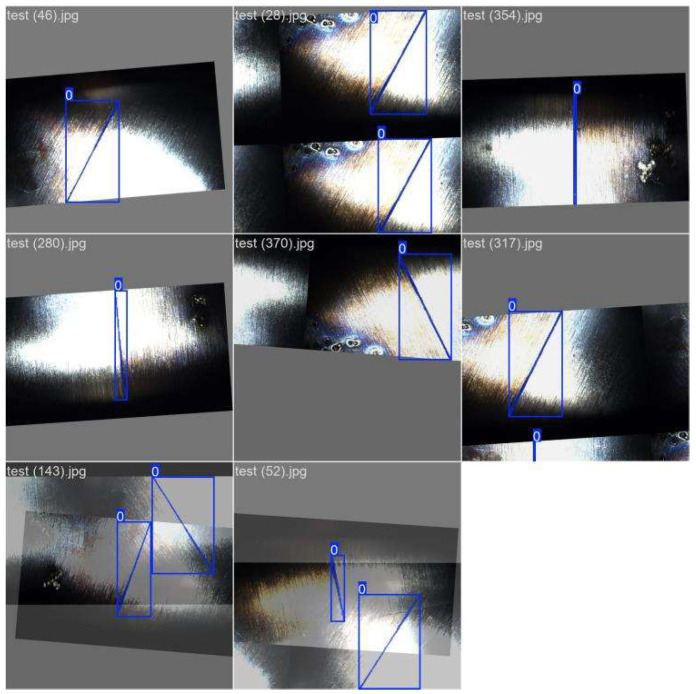
Training process example.

Following the aforementioned multi-stage enhancements, it is necessary to conduct a validation of the model’s training and performance. Figure 6 depicts the training process and its loss curve.

**Figure 7:**
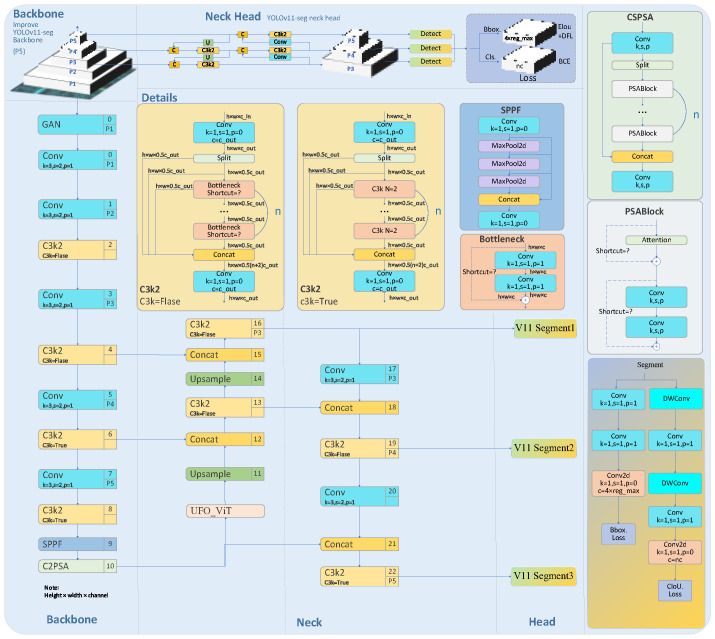
Improved YOLOv11-seg network architecture diagram.

The final modified YOLOv11-seg network architecture is shown in Figure 7.

### 2.3. Fine Positioning of Weld Seam by Adaptive Canny

The adaptive threshold Canny algorithm serves as a post-processing refinement module, operating directly on the output of the YOLOv11-seg network. This stage utilizes the coarse detection results—specifically, the predicted bounding box (bbox) and binary segmentation mask—to guide a precise, localized edge search. First, the bbox defines a Region of Interest (ROI), which is cropped from the original grayscale image. The binary mask is then applied within this ROI to isolate pixels with a high probability of belonging to the weld seam, effectively filtering out background noise. Finally, the adaptive Canny operator is applied exclusively within this masked region to extract the weld edge with sub-pixel accuracy.

This edge detection algorithm improves upon the traditional Canny method by automatically determining high and low thresholds, thereby eliminating the subjectivity and limitations inherent in manual thresholding [15]. The process commences with the application of Gaussian filtering to the original grayscale image in order to attain smoothing and reduce noise. Equation (13) represents the mathematical expression of the convolution between the Gaussian kernel and the image [16,17,18].

(13) $$G(x,y)=\frac{1}{2\pi \sigma^{2}}\;e^{-\frac{x^{2}+y^{2}}{2\sigma^{2}}}$$

Subsequently, the Sobel operator is employed to compute the gradient magnitude and direction of the image, which are, respectively, represented by Formulas (14) and (15).

(14) $$M(x,y)=\sqrt{G^{2}+G_{y}^{2}}$$

(15) $$\theta (x,y)=\arctan\frac{G_{y}}{G_{x}}$$

A non-maximum suppression procedure is used to refine the edges along the gradient direction. The histogram of image gradient amplitudes is then used to adaptively calculate high and low thresholds. The maximum entropy approach and the maximum inter-class variance method (also known as the Otsu method) are often employed techniques [19]. This work uses the Otsu approach, which uses the following Formula (16) to find the ideal segmentation threshold: t is the threshold, ω1 and ω2 are the weights of foreground and background, respectively, and μ1 and μ2 are their average gray values.

(16) $$\sigma_{B}^{2}(t)=\omega_{1}(t)\omega_{2}(t)[\mu_{1}(t)-\mu_{2}(t){]}^{2}$$

**Figure 8:**
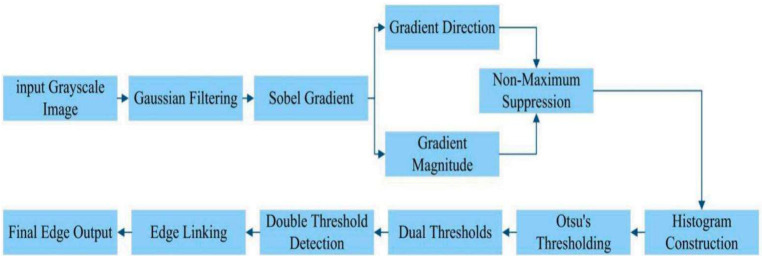
Flowchart of the adaptive threshold Canny edge detection algorithm.

The adaptive threshold Canny edge detection algorithm’s general flowchart is displayed in Figure 8.

### 2.4. Physical Coordinate Transformation and Trajectory Fitting

**Figure 9:**
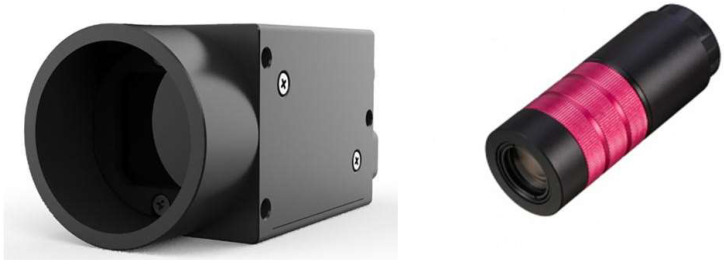
Medway MVGE800C and Dehong Technology DH250-B31.

The weld contour in the pixel coordinate system needs to be converted to the physical coordinate system of the electron beam workspace following edge fine placement [20]. The China Shenzhen Dehong Technology DH250-B31 Series Zoom Lens and China Shenzhen Medway MVGE800C series industrial surveillance cameras are depicted in Figure 9.

The spatial mapping relation between the camera and the electron beam deflection system is obtained by calibration, and the affine transformation model is established to realize the accurate conversion of pixel coordinates to physical coordinates. The internal parameter matrix after calibration is expressed by Formula (17).

(17) $$K=\left(\begin{array}{ccc} 51240 & 0 & 1864.5\\ 0 & 51480 & 1409.1\\ 0 & 0 & 1 \end{array}\right)$$

The pixel coordinates of the weld centerline’s starting and ending points, obtained through fitting, are transformed into physical coordinates via this mapping relationship. Finally, based on parameters from the welding process database (e.g., welding speed F, beam, focus, etc.), a complete G-code program containing instructions like G00 (rapid positioning) and G01 (linear interpolation) is automatically generated, achieving seamless conversion from visual information to drive commands [21].

**Figure 10:**
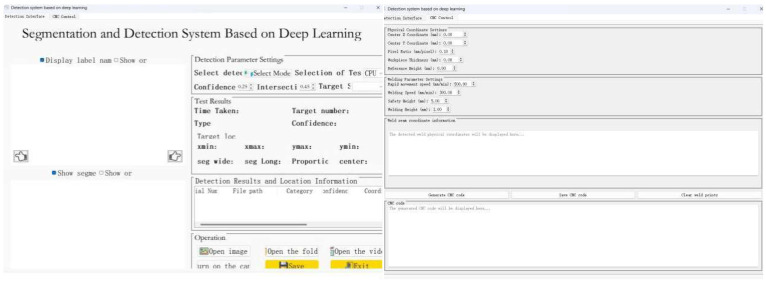
The interface of the weld G-code generation software.

The final software interface designed by the version 5.15.2 of PYQT5 [22] is shown in Figure 10.

## 3. Experimental Verification and Result Analysis

### 3.1. System Hardware Platform

**Figure 11:**
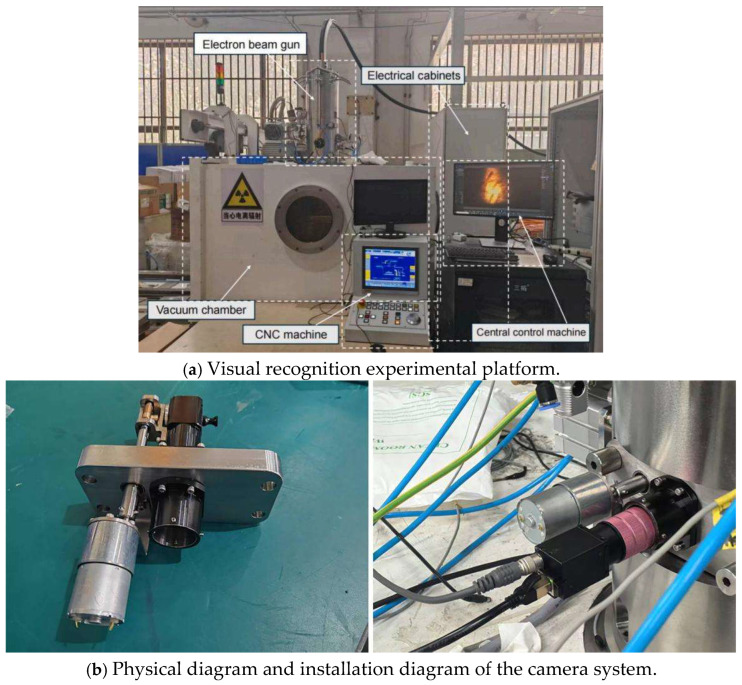
Experimental platform and physical installation diagram.

The core structure of the system is shown in Figure 11.

The system comprises a vacuum chamber, electron gun assembly, electrical cabinet, industrial PC, and CNC control unit. The machine vision system, serving as the core component, is integrated into the electron gun’s interior as shown in Figure 11. This system consists of a Medway MVGE800C industrial camera, a Dehong Technology DH250-B31 zoom lens, a custom dual-band LED light source, and a 45° refractive prism made of fused silica. This work used a prism-based refractive optical route design to accomplish visual penetration so that electron beam welding could be observed in vacuum conditions. By carefully designing the objective-prism optical system, the imaging optical route experiences two total reflections, therefore expanding industrial cameras’ field of view to the vacuum chamber’s welding region. Snell’s Law (Equation (18)) and geometric optics formulae (Equation (19)) were used to compute the angle.

(18) $$\theta_{ax}=\arcsin\left(\frac{nar}{n_{qxxw}}\sin\theta_{m}\right)$$

(19) $$d=f_{obi}+L_{ax}+\Delta$$

The following specs were obtained from the calculations: The objective lens working surface is located 144.5 mm from the prism’s incident surface, and the prism has a 45-degree right-angle optical arrangement with optimized dimensions of 20 mm × 20 mm × 20 mm. The prism holder, which has a modular design, is firmly attached to the vacuum flange in order to satisfy the vacuum chamber’s operating needs as well as visual inspection criteria. This integrated design guarantees adherence to vacuum chamber performance requirements as well as visual inspection standards.

### 3.2. Algorithm Experiment

#### 3.2.1. Algorithm Experiment Settings

**Figure 12:**
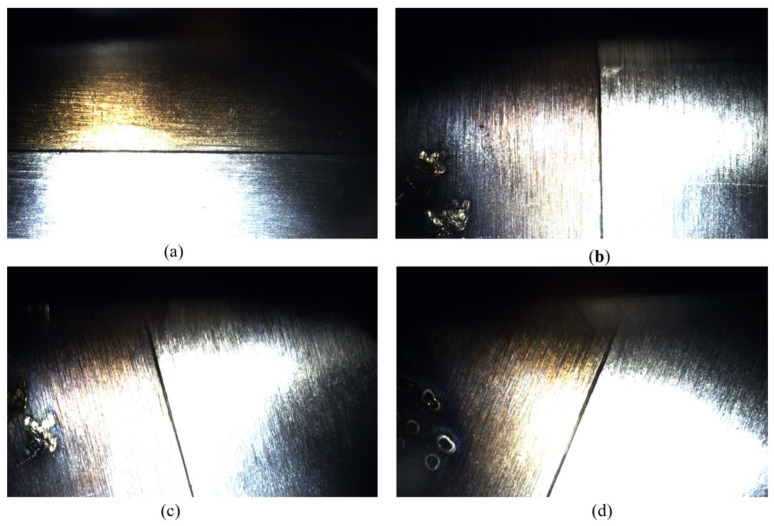
Partial images of the dataset. Note: (**a**) 0° side seam; (**b**) turn right 90°; (**c**) turn left 70°; (**d**) turn right 70°.

**Figure 13:**
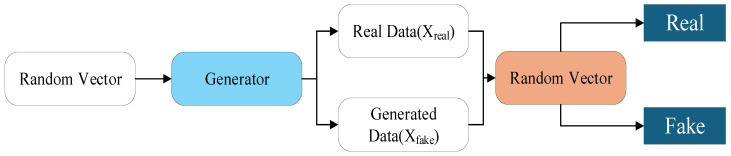
GAN generates an adversarial network architecture.

The experiment was conducted on a 1 m^3^ vacuum chamber platform. The dataset comprises 618 autonomously captured stainless steel weld images (Table 1), covering multiple orientations including horizontal, 90° right turn, 70° left turn, and 30° left turn, with selected images shown in Figure 12. To enhance model generalization, a Generative Adversarial Network (GAN) was employed exclusively for data augmentation of the training set (Figure 13) [23,24,25], expanding it from 618 to 1300 images. The validation and test sets contained only original, non-synthetic images to ensure an unbiased evaluation. The software environment utilized PyTorch 2.2.2 and Python 3.10.12, while the hardware platform featured an Intel i9-13900HX CPU and NVIDIA RTX 4060 GPU.

#### 3.2.2. Evaluation Indicators

In deep learning, evaluation metrics such as precision, mean average precision (mAP), and recall rate are used to assess network performance [26,27]. Formula (20) represents the recall rate.

(20) $$\mathrm{Recall}=\frac{\mathrm{TP}}{\mathrm{TP}+\mathrm{FN}}$$

Formula (21) is used to express precision, where TP, FN, and FP represent the number of successfully identified weld points (TP), missed weld points (FN), and false positive weld points (FP), respectively.

(21) $$Precision=\frac{TP}{TP+FP}$$

Formula (22) is used to represent the F1 score.

(22) $$F1=\frac{2\times Precision\times Recall}{Precision+Recall}$$

Formula (23) represents mAP.

(23) $$mAP=\frac{\sum_{n=1}^{N}AP_{n}}{N}$$

The detection precision for a particular target category is indicated by mean accuracy (AP) in the mean accuracy (mAP) measure, where N is the total number of target categories. The evaluation criterion is the Intersection-Union (IoU) ratio threshold: a detection is considered valid if the ground truth bounding box and the predicted bounding box overlap by at least 50% (i.e., IoU ≥ 0.5).

#### 3.2.3. Comparative Tests

To scientifically evaluate the performance and industrial applicability of the proposed electron beam weld seam recognition system, this study designed a comparative experiment against several widely adopted baseline models in practical machine vision: the YOLOv11 algorithm, the YOLO-v8 algorithm, and the Faster R-CNN algorithm. These models were selected because they represent the two predominant and industrially relevant detection paradigms—single-stage (YOLO series) and two-stage (Faster R-CNN) detectors—commonly deployed or evaluated in real-world production settings.

To scientifically evaluate the performance of the electron beam weld seam recognition system based on the improved YOLOv11-seg visual algorithm, this study designed a comparative experiment to evaluate its performance against the YOLOv11 algorithm, Faster R-CNN algorithm, and YOLO-v8 algorithm.

We developed a multi-dimensional evaluation framework, with key metrics including mean accuracy (mAP), training duration, F1 score, and model size. It is important to note that these metrics should be evaluated in their actual application contexts. In specialized industrial environments and high-dynamic interference scenarios, the effective range of mean accuracy is typically between 50% and 75%. For a more comprehensive data comparison, please refer to the quantitative analysis results presented in Table 2.

The analysis of the table above demonstrates that the improved YOLOv11-seg model proposed in this study significantly outperforms the benchmark model in both mAP and F1 scores, achieving 78.6% and 77.8%, respectively, which validates its superior performance in welding seam recognition. Despite its slightly larger size, the model achieves a frame rate of 20 fps, meeting real-time requirements, with training duration kept within a reasonable range, resulting in overall optimal performance. Additionally, the improved model exhibits outstanding robustness under complex texture interference and low-contrast conditions, effectively enhancing defect recognition accuracy during electron beam welding. Further validation through ablation experiments is shown in Table 3.

Experimental results demonstrate that both the GAN integration and the UFO_ViT module significantly enhance model performance. Notably, our comprehensive approach achieves a 13.2-percentage-point improvement in mean area precision (mAP) over the baseline YOLOv11-seg model, validating the effectiveness and synergistic benefits of the proposed modules.

### 3.3. Experiment of Weld Track Generation

#### 3.3.1. Comparative Experiments

**Figure 14:**
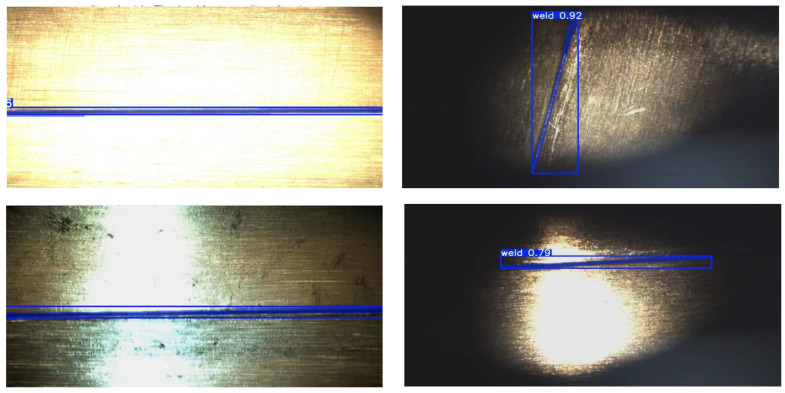
Model recognition performance under different illumination conditions.

In order to verify the accuracy and stability of the weld track generation, four sets of welds under different illumination are used for the recognition and generation experiment, and the results are shown in Figure 14.

The model can accurately capture the weld boundary and generate smooth trajectory curve under the background of strong light, weak light and complex reflection, showing good adaptability and robustness. The accuracy of the captured boundaries and generated trajectories was quantified by comparing them against manually annotated weld centerlines, which served as the ground truth for measurement. Especially in a low illumination environment, the model can maintain higher geometric consistency and positioning accuracy than the traditional method.

**Figure 15:**
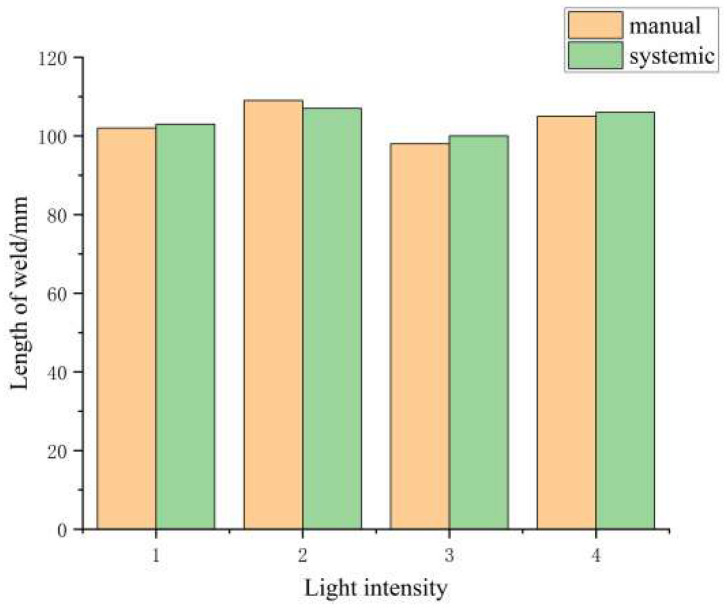
Comparison of weld recognition and trajectory generation errors.

The results of the quantitative analysis are shown in Figure 15.

As shown in Figure 15, the proposed method achieves the lowest average positioning error and trajectory deviation (both under 3 mm) across all lighting conditions, with a significantly faster processing time than manual methods.

#### 3.3.2. Experiment of Weld Track Generation and Code Generation

After weld recognition, the system generates continuous weld trajectories from the identified results, converts them into executable motion paths, and produces corresponding G-code. In this step, we randomly select weld images and require that the system generate continuous weld trajectories based on the recognition results, then fit them into smooth curve paths.

**Figure 16:**
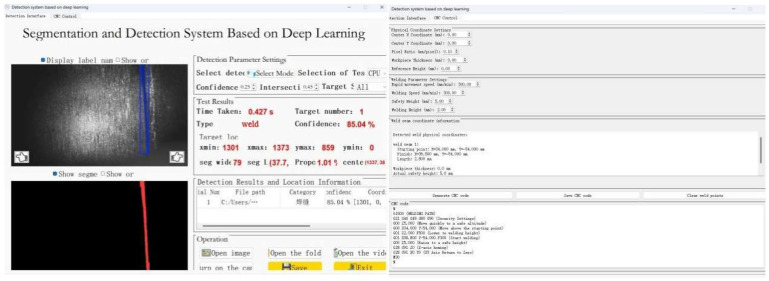
Code generation and actual weld shape recognition.

The fitted path is then mapped to the physical coordinate system via the coordinate transformation module, and G-code is automatically generated by integrating preset welding parameters. Some results are shown in Figure 16.

The experimental results show that the generated G-code trajectory is highly consistent with the actual weld shape, and the path continuity is good, without obvious jump or break point.

## 4. Conclusions

To address the need for manual intervention in weld positioning during electron beam welding, this study proposes a deep learning-based weld recognition system. The system innovatively integrates visual sensors into the welding testing platform, achieving a fully closed-loop detection process from image acquisition to trajectory planning. Experimental results demonstrate that the system maintains 77% recognition accuracy and 19 FPS recognition speed even under interference conditions, meeting real-time requirements with a 9% improvement over the baseline model. Key conclusions include the following:

(1) A specialized weld image dataset for vacuum high-reflection environments was constructed, featuring algorithmic and hardware-level optimizations to effectively suppress metallic reflections, motion blur, and other high-dynamic interference.
(2) A hybrid algorithm integrating YOLOv11-seg and adaptive Canny edge detection was proposed to achieve real-time robust weld seam recognition under computational constraints.
(3) Through the integration design of an industrial camera and optical prism, the visual penetration and stable imaging of welding area in a vacuum environment are realized, which provides a high-quality input source for subsequent recognition.

This study has demonstrated the feasibility of the proposed vision system for straight stainless-steel weld recognition in vacuum environments. However, the work acknowledges critical limitations in weld diversity and material applicability. The current validation, focused predominantly on linear weld forms and a single material (stainless steel), constrains the system’s broader industrial relevance. To bridge this gap and enhance adaptability for complex welding scenarios, future work will prioritize three directions: (1) expanding the material library to include titanium and aluminum alloys with varied surface treatments; (2) developing robust recognition architectures for curved and multi-pass welds; and (3) exploring multi-sensor information fusion, particularly integrating infrared vision, to achieve synergistic monitoring beyond the visible spectrum.

## Figures and Tables

**Table 1:** Comparison of image quantity and type for each dataset section.

Class	Side Seam	Turn Right 90°	Turn Left 70°	Turn Right 70°	Amount to	Material
Train	85	206	149	60	500	nonrust steel
Val	10	23	17	9	59	nonrust steel
Test	19	13	15	12	59	nonrust steel

**Table 2:** Experimental comparison of different models.

Method	mAP (%)	FPS	Epochs	Model-Size/M	F1-Score (%)
YOLOv11	68.3	18	400	7.2	72.1
Faster R-CNN	66.1	6	400	330.2	67.4
YOLO-V8	67.2	15	400	5.2	69.9
Ours	78.6	20	400	8.5	77.8

**Table 3:** Algorithm ablation experiment.

Method	mAP (%)	F1-Score (%)	ΔmAP (vs. YOLOv11-Seg)
YOLOv11-seg	69.4	72.1	0
YOLOv11-seg + GAN	74.3	75.3	+7.1%
YOLOv11-seg + UFO_ViT	72.8	74.3	+4.9%
YOLOv11-seg + EIOU	72.5	73.9	4.5%
Ours	78.6	79.2	+13.2%

## Data Availability

The dataset utilized in this study is proprietary. It is available from the first author upon reasonable request for purposes of academic validation and non-commercial research.
